# Supplementary material for: 18Beta-Glycyrrhetinic Acid Attenuates H2O2-Induced Oxidative Damage and Apoptosis in Intestinal Epithelial Cells via Activating the PI3K/Akt Signaling Pathway
Source: Antioxidants (Basel). 2024 Apr 16;13(4):468. doi: 10.3390/antiox13040468 (PMC11047483; doi:10.3390/antiox13040468)
Supplement: Supplementary file 1 [file antioxidants-13-00468-s001.zip › antioxidants-2889724-supplementary.pdf]

Glycyrrhetinic Acid Attenuates H_2O_2-Induced Oxidative Damage and Apoptosis in Intestinal Epithelial Cells via Activating the PI3K/Akt Signaling Pathway

Cui Ma[a], Fuxi Wang[a,d], Jiawei Zhu[a], Shiyi Wang[a,c], Yaqing Liu[a,b], Jianfang Xu[a], Qingyu Zhao[a], Yuchang Qin[a], Wei Si[a*], and Junmin Zhang[a*]

[a] State Key Laboratory of Animal Nutrition and Feeding, Institute of Animal Sciences of Chinese Academy of Agricultural Sciences, Beijing, 100193, China.

[b] College of Animal Science and Technology, Qingdao Agricultural University, Qingdao, 266109, China.

[c] College of Food Science and Engineering, Qingdao Agricultural University, Qingdao, 266109, China.

[d] College of Animal Science and Technology, Shanxi Agricultural University, Taigu, Shanxi 030801, China

[*] Correspondence: Dr. Wei Si, siwei01@caas.cn
[*] Correspondence: Dr. JunMin Zhang, zhangjunmin@caas.cn

Supplementary Figure S1 Effect of hyperoxia and hydrogen peroxide (H_2O_2) and glycyrrhetinic acid (GA) on cell viability in IPEC-J2 cells. (A) Cell viability in IPEC-J2 cells exposure to 200 uM H_2O_2 for 8 h. (B) Cell viability in IPEC-J2 cells pre-treated with GA for 24 h exposure to 200 uM H_2O_2 for 8 h. Data are presented as mean ± SEM with triplicate cultures of the representative experiments. (A) Data were analyzed by unpaired T-test. *$p < 0.05$ means significant difference between two groups.

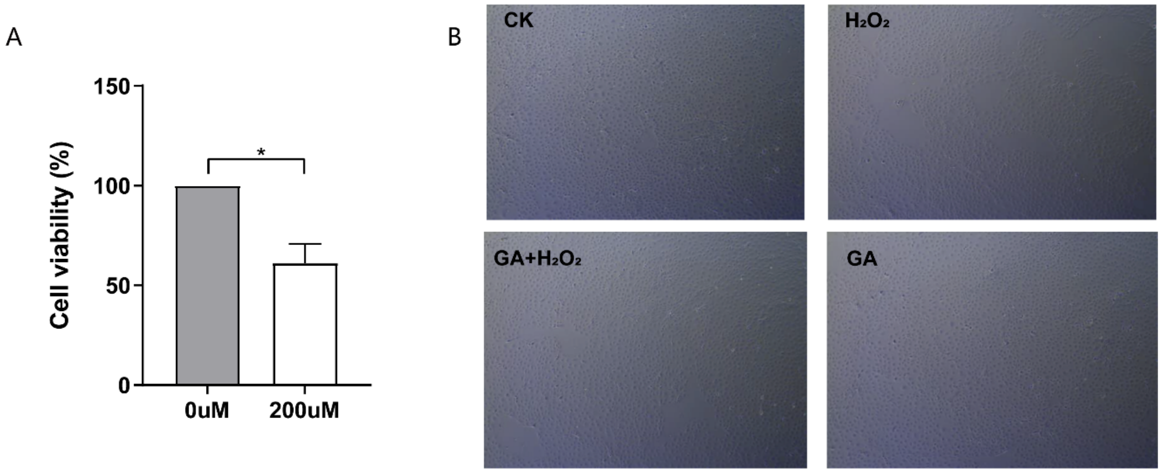

Supplementary Figure S2 The western blot images of p-PI3K, PI3K, p-Akt, and Akt were run in parallel and left and right panels were run at different times.

updated Figure S2

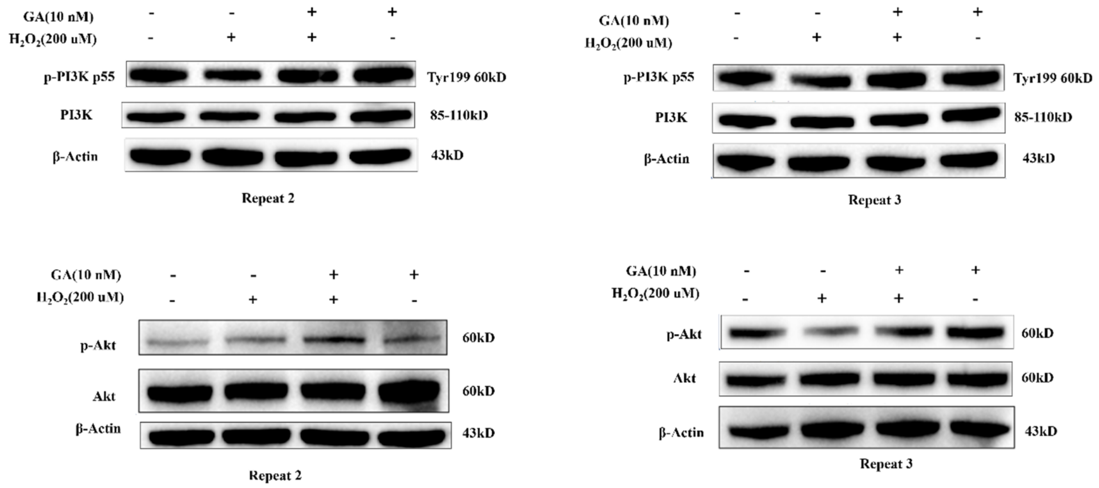

Supplementary Figure S3 The western blot images of BCL2, BAX, p53, and Cytc were run in parallel and left and right panels were run at different times.

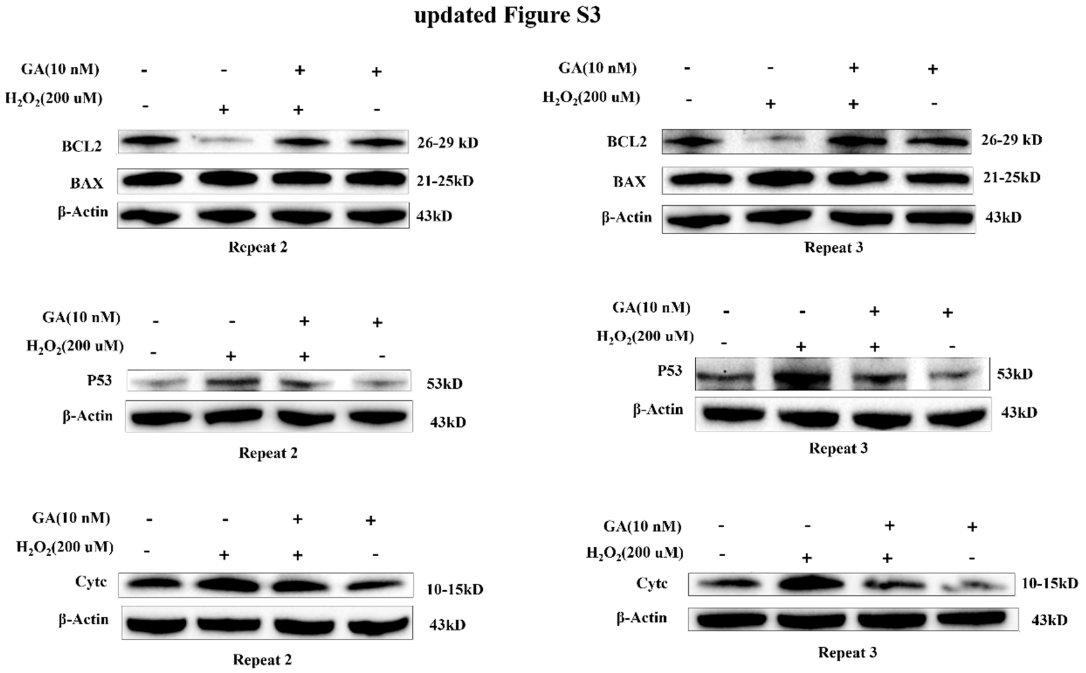

Supplementary Table S1 Information of the commercial assay kits

Index	Item number	Company
MDA	A003-1-2	Nanjing Jiancheng Bioengineering Institute
SOD	A001-1-2	Nanjing Jiancheng Bioengineering Institute
GSH/GSSG	A061-2-1	Nanjing Jiancheng Bioengineering Institute
CAT	A007-1	Nanjing Jiancheng Bioengineering Institute

Supplementary Table S2 The raw data of MDA level, SOD and CAT activity, and GSH/GSSG ratio

Group	MDA (nmol/10^6 cell)	SOD (U/10^6 cell)	CAT (U/10^6 cell)	GSH/GSSG
CK	0.83	8.02	1.48	2.66
CK	0.96	7.56	0.70	2.51
CK	0.74	3.38	1.00	1.57
CK	0.79	3.47	0.70	2.68
CK	0.99	8.65	1.58	1.51
CK	0.99	8.56	1.45	1.7
CK	0.74	3.61	0.71	1.55
CK	0.76	8.54	1.46	2.95
CK	0.97	3.63	0.72	2.55
H_2O_2	1.20	7.10	0.65	2.25
H_2O_2	1.32	6.65	0.64	2.55
H_2O_2	1.27	6.60	0.64	2.38
H_2O_2	1.31	7.20	0.65	2.47
H_2O_2	1.30	6.64	0.64	2.7
H_2O_2	1.34	6.60	0.64	2.56
H_2O_2	1.34	7.13	0.65	2.18
H_2O_2	1.21	7.15	0.65	2.64
H_2O_2	1.20	7.10	0.64	2.5
GA+ H_2O_2	0.99	7.40	2.12	3.75
GA+ H_2O_2	1.02	7.48	1.60	4.09
GA+ H_2O_2	0.95	6.79	2.10	2.72
GA+ H_2O_2	1.03	6.80	2.18	4.44
GA+ H_2O_2	1.03	7.60	1.60	4.14
GA+ H_2O_2	1.03	7.57	1.64	4.3
GA+ H_2O_2	0.95	6.81	2.17	4.59
GA+ H_2O_2	1.03	6.85	1.60	3.52
GA+ H_2O_2	0.97	7.57	2.22	2.9
GA	0.81	7.63	1.49	1.95
GA	0.89	8.10	2.27	3.97
GA	0.80	7.07	1.12	2.53
GA	0.81	8.28	1.30	1.98
GA	0.80	8.30	2.50	2.61
GA	0.90	7.13	2.44	2.02
GA	0.90	7.22	1.15	1.86
GA	0.91	7.54	1.23	3.96
GA	0.81	8.16	1.27	2.59

Supplementary Table S3 Primer sequences of genes for quantitative real-time PCR

Gene	Primer sequence (5′-3′)	Accession number	Size (bp)
GAPDH	F: TGTCCACCTTCCAGCAGATGT	NM_001206359.1	132
	R: AGCTCAGTAACAGTCCGCCTAGA		
iNOS	F: GGGTCAGAGCTACCATCCTC	XM_013981169.2	114
	R: CGTCCATGCAGAGAACCTTG		
CP	F: TGGAGCCAGACAATGAAGAC	NM_001267694	160
	R: CATCAAAGAGGGTAGCAGGA		
VIPR1	F: CATCAGGCTGCAGCAAGATGT	NM_214036.1	219
	R: CGTCCAAACCCGATGCCTTGTC		
ZNF554	F: CCGGGAACCCAGAGCCTTAT	XM_021084174.1	203
	R: CAGCCACATCCTCCAAGGTT		
P53	F: CTGGCAGCAGTGAACGATCT	NM_213824.3	148
	R: TGCAGGAACCCTAGACGGAA		
CytC	F: AGACTGGTCCAAACCTCCAT	NM_001129970.1	190
	R: TCTCCCTTCTTCTTAATGCCAG		

Supplementary Table S4 Information of primary antibodies

Primary antibodies	Item number	Source	Dilution rate	Company
β-Actin	4967	Rabbit	1：5000	Cell Signaling
PI3K	3811S	Rabbit	1：2000	Cell Signaling
p-PI3K	4228T	Rabbit	1：2000	Cell Signaling
Akt	9272	Rabbit	1：1000	Cell Signaling
p-Akt	9271s	Rabbit	1：1000	Cell Signaling
Bcl2	12789-1-AP	Rabbit	1：2000	Proteintech
Bax	50599-2-lg	Rabbit	1：1000	Proteintech
p53	9282T	Rabbit	1：1000	Cell Signaling
Cytochrome C	10993-1-AP	Rabbit	1：1000	Proteintech

Supplementary Table S5 Quality of sequencing data from IPEC-J2 cells samples libraries

Sample	Raw_reads	Clean_reads	Error_rate	Q20(%)	Q30(%)	GC_pct(%)
CK-1	45743280	44440402	0.03	97.43	93.11	52.45
CK-2	47727540	46627492	0.03	97.58	93.51	52.58
CK-3	45913052	44880952	0.03	97.4	93.07	52.84
H_2O_2-1	47114360	45846546	0.03	97.74	93.8	52.89
H_2O_2-2	45827002	44874892	0.03	96.67	91.11	53.46
H_2O_2-3	45909150	44394208	0.03	96.88	91.5	52.75
GA+ H_2O_2-1	42174320	41045548	0.03	97.17	92.61	52.51
GA+ H_2O_2-2	46761698	45710858	0.03	97.25	92.81	52.92
GA+ H_2O_2-3	45659170	44350230	0.03	97.02	92.26	52.9
GA-1	44964048	43955882	0.03	97.08	92.33	52.03
GA-2	47970984	46808930	0.03	97.4	93.12	52.82
GA-3	46249248	45065328	0.03	97.18	92.55	52.13
